# Comparing Aha! Moments in Problem Solving and Generative Ideation

**DOI:** 10.3390/jintelligence14080161

**Published:** 2026-08-01

**Authors:** Visheeta J. Chandolia, Matthew A. Kidd, Morgan S. Paladino, Steven M. Smith

**Affiliations:** Department of Psychological and Brain Science, Texas A&M University, College Station, TX 77840, USA; matthew.kidd@tamu.edu (M.A.K.); msp@tamu.edu (M.S.P.); stevesmith@tamu.edu (S.M.S.)

**Keywords:** problem solving, generative ideation, aha, metacognition

## Abstract

Responses that come to mind as aha! moments occur suddenly, rapidly, and without deliberate intention. Aha! moments are typically associated with insight, the sudden realization of a problem’s solution, and have recently been reported in generative tasks, including divergent thinking and category generation. The present experiment compared the frequency and strength of aha! moments for responses in verbal problem solving (Compound Remote Associates and Polysemous Associates Test problems) with responses in ad hoc and taxonomic category generation. Comparisons were made using a two-step procedure in which each response was first reported as an aha! or non-aha! response, and, for aha! responses, rated in subjective strength. No difference in aha! strength was found between problem solutions and category responses, regardless of which task was given first, nor did aha! strength differ between ad hoc and taxonomic category members. In generative tasks, aha! strength, pre-response pause time, and response novelty increased over time during generation. Although fewer responses occurred over time, later responses were increasingly likely to be aha! responses. These results support the use of aha! reports during idea generation and link such reports to retrieval dynamics seen in many generative tasks, including divergent thinking.

## 1. Introduction

Most studies of insight, that is, moments when people realize solutions to problems, have focused on problem solving in which awareness of a solution can occur suddenly, rapidly, and without deliberate intention. The subjective experience of insight is often measured by metacognitive aha! judgments after solving a problem. The aha! moment, also understood as the feeling of insight, or the sudden and unexpected realization of a solution, is traditionally studied using insight problems such as the Compound Remote Associate (CRA) problems (Bowden, 1997), matchstick problems (Knoblich et al., 2001), magic tricks (Danek et al., 2014; Hedne et al., 2016), and analogies (Chesebrough et al., 2023), among other methods. Insight is an effective problem-solving approach, often credited with enabling major breakthroughs, including Einstein’s theory of relativity (Kounios & Beeman, 2014) and Kary Mullis’s invention of PCR (Ovington et al., 2018). Furthermore, insight strengthens the consolidation of the problem and its solution in memory (e.g., M. Becker et al., 2025; Danek et al., 2013; Ludmer et al., 2011).

Similar subjective qualities of suddenness and surprise have also been studied outside of the insight problem-solving literature. For example, metacognitive reports of mind-pops (e.g., Kvavilashvili & Mandler, 2004; Mandler, 1995), such as spontaneous autobiographical memories (e.g., Berntsen, 1996, 1998, 2021), involuntary semantic memories (e.g., Kvavilashvili & Mandler, 2004), and spontaneous episodic memories (e.g., Berntsen et al., 2013), resemble the methods used to define aha! moments. Furthermore, a recent study reported that aha! moments occur during generative tasks, including divergent thinking, ad hoc category generation, and taxonomic category generation (Smith et al., 2025a). In the present study, we compared subjective reports of aha! moments in a generative task (listing members of taxonomic and ad hoc categories) with those found when people solve verbal insight problems, which are considered by many researchers to be the “gold standard” of insight problems.

The insight experience has been attributed to several explanations, including the remote association theory (Mednick, 1962) and the restructuring hypothesis (Ohlsson, 1992). The remote association theory suggests that insight occurs when individuals form connections between distantly related concepts in memory, enabling a solution that was not initially accessible through more obvious or dominant associations (Mednick, 1962). According to Mednick’s associative theory of creativity, ideas in memory are organized into associative hierarchies, in which common associations are more easily retrieved than remote ones (Mednick, 1962). Tasks such as the Remote Associates Test (RAT, e.g., Mednick, 1962) were developed to measure the ability to identify a single concept linking several seemingly unrelated cues, reflecting the cognitive process of retrieving remote associations in creative problem solving (Marko et al., 2019). The restructuring hypothesis emphasizes changes in the mental representation of a problem rather than the retrieval of distant associations (Wertheimer, 1959). It suggests that insight problems initially lead individuals to form an incorrect or constrained representation of the problem, resulting in an impasse during the problem-solving process. Insight occurs when the solver restructures or reinterprets the problem representation, allowing previously overlooked relationships or solution paths to become apparent (Ohlsson, 1992; Ash & Wiley, 2006). This process can involve relaxing inappropriate constraints, shifting attention to previously ignored aspects of the problem (Knoblich et al., 2001), or reorganizing elements of the problem in a new way. Empirical research supports this view by demonstrating that successful insight solutions are often preceded by changes in attention patterns and problem representation, indicating a restructuring of the cognitive representation underlying the problem (M. Becker et al., 2025; Knoblich et al., 2001). Both accounts entail a feeling of being “stuck” or fixated. The sudden and unexpected resolution of this fixation by accessing a remote associate or restructuring the problem representation marks an insight.

It is important to note the distinction between theoretical accounts of insight and the methods used to measure insight. The aha! moment is assumed to accompany insight, but they may not always reflect the same thing (Wiley & Danek, 2024). Insight is defined as a cognitive process that typically follows an impasse that is resolved via restructuring (Topolinski & Reber, 2010). Aha! moments are a metacognitive component of an insight, characterized by feelings of suddenness, correctness, confidence, and delight (Danek & Wiley, 2017). An aha! moment seems to pop into one’s mind, typically after a long pause (Salvi et al., 2016) or upon solving a problem following unsuccessful solution attempts (Ohlsson, 1992; Danek et al., 2014). However, several studies use the terms interchangeably (e.g., Bowden & Jung-Beeman, 2003; Kounios & Beeman, 2009, 2014). These studies relied on the a priori classification of certain problems (like rebus problems or the nine-dot problem) as insight problems (Weisberg, 1995; see also Webb et al., 2016). However, there is some evidence that challenges the assumption that insight and aha! moments are one and the same. For instance, Danek and Wiley (2017) showed that aha! moments may sometimes accompany an incorrect response, suggesting that an aha! is not a definitive indicator of a successful insight. Furthermore, some aha! moments may not be underscored by an insight and instead could be a product of a solution flooding the consciousness quickly (Topolinski & Reber, 2010).

### 1.1. Metacognitive Reports of Insight

Research on insight in creative problem solving relies on an underlying assumption that problems are solved through either an analytic approach, in which participants can report the steps leading to a solution, or through a process of sudden restructuring of mental representations, often accompanied by the feeling of an aha! (Sternberg & Davidson, 1995). Studies have used various means to record aha! moments during problem solving, such as a self-report paradigm, wherein participants report if they had an aha! moment after they solve each problem (Aziz-Zadeh et al., 2009; Bowden & Jung-Beeman, 2003; Kounios & Beeman, 2015). Some studies have used a think-aloud method to trace when participants realize a solution (Fleck & Weisberg, 2013; Schooler et al., 1993), showing that participants stop verbalizing before they have an insight moment, and that verbalizing impaired performance, particularly when solving problems with insight as opposed to analytical solving.

An insight typically follows the successful resolution of an impasse. The impasse is often measured using self-reports (e.g., Beeftink et al., 2008; Danek et al., 2014; Webb et al., 2016; Shen et al., 2019) and behavioral markers (e.g., Fleck & Weisberg, 2013; Fedor et al., 2015). However, there is growing evidence that participants self-report fewer impasses than are indicated by behavioral measures, raising questions about the reliability of self-reports of impasses (Ross & Arfini, 2024). Restructuring during problem solving is inferred following the sudden and successful resolution of an impasse (e.g., Metcalfe & Wiebe, 1987; Ash & Wiley, 2006), when solving a problem requiring an alternative or unusual representation (e.g., CRA and PAT problems, and classical insight problems like the nine-dot problem), or upon overcoming priming-induced fixation (Storm et al., 2011).

A pioneering study by Metcalfe and Wiebe (1987) used subjective warmth-to-solution ratings for classical insight problems. Unlike analytical problem solving, in which participants gradually moved from cold (very far from solving the problem) to warm (close to solving the problem), insight moments were accompanied by a sudden shift from cold to warm right before a solution occurred (Metcalfe & Wiebe, 1987).

While several studies report a binary measure of aha! (i.e., whether or not participants experienced an aha! moment during problem solving), some studies use graded judgements to better understand nuances in insight problem solving. Some graded metacognitive judgments of insight include reports of confidence in a solution, pleasure, strength of an aha!, suddenness of solution, and feelings of impasse and surprise (Danek et al., 2015; Sandkühler & Bhattacharya, 2008). Hedne et al. (2016) found that confidence is higher for insight solutions compared to non-insight solutions, and that this confidence is strongly correlated with accuracy for insight solutions. Furthermore, the aha! experience is associated with feelings of delight upon solving the insight problem (Danek et al., 2015). Numerous explanations of this associated delight exist, such as positive self-evaluations following the solution (Danek et al., 2015), due to a prediction error (Savinova & Korovkin, 2022), or due to reinforcement following the solution (Danek & Wiley, 2020), among other explanations.

Graded measures of insight have also been used to capture variance in the intensity or strength of an aha! experience (Webb et al., 2016; Chesebrough et al., 2023). Strength is an interesting and important feature of the aha! moment, as it is associated with several outcomes. For instance, aha! strength is associated with greater accuracy (Salvi et al., 2016), higher ratings of pleasure (Danek et al., 2015), higher confidence (M. Becker et al., 2025; Chesebrough et al., 2023) and preceded by stronger feelings of impasse (Webb et al., 2016). Finally, aha! strength has been observed to have a curvilinear association with problem difficulty, with items of moderate difficulty exhibiting the highest strength (Chesebrough et al., 2023).

Several factors affect the reported strength of an aha! moment. For instance, a self-generated solution is associated with a stronger aha! (Mori, 1996). The same study found that a stronger aha! is reported when participants dwelled on possible solutions for longer before being presented with the correct answer (Mori, 1996). Aha! moments were also stronger in a no-hint condition compared to when hints were given when solving anagrams (Ammalainen & Moroshkina, 2021). Problem type may also determine aha! strength. In a study analyzing the strength of aha! ratings across several types of insight and non-insight problems, anagrams were found to be associated with the highest accuracy and aha! strength (Webb et al., 2018). Although CRA problems were also solved with the highest aha! strength among the various types of insight problems compared, the solution rates for CRA were lower than for anagrams. Webb et al. (2018) explained these results as arising from the short solution times and unambiguous one-word solutions in these problems. Participants in their study reported some aha! moments for inaccurate solutions, but these false insights tended to be weaker than those for correct solutions. Overall, Webb et al. (2018) found that insight moments are reported across problems, irrespective of whether they are traditionally insight problems or not, but insights are stronger and more frequent during classical insight problems, followed by CRA problems, and non-insight problems. This prompts a study into the direction of using tools other than insight problems and binary judgments of insight to better understand aha! moments.

An important methodological consideration when using graded measures of aha! strength is the inclusion of a binary (yes/no) report of the aha! moment before collecting graded judgments of related outcomes such as suddenness, strength, and confidence. Whereas some studies collect a binary report first (e.g., Danek & Wiley, 2017; Danek et al., 2014), most studies use a continuous rating scale that includes 0 (Chesebrough et al., 2023; Salvi et al., 2016; Webb et al., 2016; Webb et al., 2019). However, the recognition literature points to the idea that a total absence of an internal signal (“0”) as the baseline of a continuous spectrum introduces response biases (Rotello et al., 2005; Wixted & Mickes, 2010), emphasizing that the absence of an experience is qualitatively distinct from a low-strength experience. In our study, we use a two-step measure where we first collect a binary judgment about whether a response or idea was an aha! moment. If an aha! was reported, then a graded judgment of how strong or intense the aha! felt was collected on a scale of 1 (“very weak aha!”) to 5 (“very strong aha!”). A novel methodological contribution of our study is the direct comparison of standard measures of aha! outcomes (0–5) with our two-step measure (1–5).

### 1.2. Insight Moments in Idea Generation

In the creative cognition literature, insight problems are considered a convergent thinking task, that is, the cognitive process of generating a single correct solution (Seifert et al., 1994). In contrast, a divergent thinking task prompts participants to produce many different ideas, like during brainstorming (Osborn, 1957). Rather than accuracy or insight experiences, performance is usually measured by the creativity, novelty, and fluency of responses, such as in the alternative uses task (Guilford, 1967). To investigate different types of creative thinking, these two paradigms have historically operated independently of each other (Zhang et al., 2020), as they are often purported to involve separate cognitive processes—convergent thinking for typical insight problems, and divergent thinking for alternative uses task type of problems.

However, this assumption has been recently challenged (Eymann et al., 2026). Emerging studies are pointing to a unified view of creative generative ideation that suggests responding to insight problems and divergent thinking problems entails the same underlying process of idea generation (Smith et al., 2025a). First, participants generate potential solutions or ideas for a problem, often reaching for the most common dominant responses. Then, these dominant responses build output interference, preventing generation of new ideas and causing fixation. This is followed by restructuring, where a new interpretation of the problem leads to a new idea, often accompanying an insight experience. There is growing evidence supporting this idea; for example, in a previous study, we found that participants reported insight experiences during a divergent thinking task (Smith et al., 2025a). Furthermore, they reported insight moments during taxonomic category generation (names of countries, fruits, etc.) and ad hoc category generation (things that are green, that make noise, etc.) tasks (Smith et al., 2025a). Moreover, responses reported as aha! moments were more creative and novel than non-aha! responses.

The model suggests that instead of viewing convergent and divergent thinking processes as distinct, they are more simply explained by the process of generating multiple ideas, experiencing fixation in response to generating multiple ideas, and then overcoming said fixation to identify the new solution (Smith et al., 2025a). This can work for convergent thinking problems like the CRA, where participants start by listing the most common associates to the problem words, until they are fixated, and then suddenly realize the correct remote associate. This works similarly for divergent thinking problems; for instance, one can start with common uses of a brick, until they are fixated and cannot think of a novel use. If cognitive restructuring occurs, they might produce a new idea and likely report this as an insight. Thus, we propose that the conceptualization and independent investigation of convergent thinking and divergent thinking are ineffective. Rather, we endorse the utilization of these two tools (i.e., problem solving and idea generation) to explore the cognitive process of producing creative ideas, specifically those that come about from an aha! moment.

Until recently, aha! moments have been explored exclusively with a problem-solving paradigm. Others have found that aha! moments are associated with a feeling of suddenness, delight, and correctness (Danek et al., 2014). Several studies also include restructuring as an essential aspect of insight, such that it is not the type of problem itself, but the sudden restructuring that precedes the realization of the solution, that causes the aha! (Fleck & Weisberg, 2013). Other explanations of the aha! rely on the fluency hypothesis, suggesting that when a bulk of information floods into consciousness, it is experienced as an aha! (Topolinski & Reber, 2010).

### 1.3. The Nascent Pause

The nascent pause refers to the period preceding an insight where cognitive restructuring might occur. The nascent period in a generative task manifests as a 6.5 s greater pause time in aha! responses compared to non-aha! (Smith et al., 2025a). Several explanations in the insight problem-solving literature have been proposed to explain it. One account pertains to the switch from a verbalizable mode of thinking to a non-verbalizable mode of thinking during the nascent period, as found in a think-aloud protocol during an insight problem-solving task (Schooler et al., 1993). Additionally, Beaty et al. (2014) have posited a functional network related to divergent thinking that involves coupling of the brain’s default mode network (DMN) and executive control network (ECN) and becomes more active over the course of a divergent thinking task. Another explanation entails the interplay between the DMN and ECN, where a shift from DMN to ECN activity may signal the transition from idea generation to evaluation and selection of ideas (Chen et al., 2025; Webb et al., 2019). Additionally, the exploration–exploitation trade-off, which refers to the balance between exploring new ideas and exploiting existing knowledge, may play a role in shaping the dynamics of the nascent period (Ovando-Tellez et al., 2025). A study found that increased eye-blink rate and pupil constriction are associated with the exploitative process of “convergent thinking”, while slower eye-blink rate and pupil dilation are associated with exploration as seen in “divergent thinking”. Eye blink and pupil dilation were found to be significantly associated with flexibility during “divergent thinking”, and with accuracy in “convergent thinking” (Chermahini & Hommel, 2010). In the current study, the nascent pause serves as an observable behavior that may reflect underlying cognitive processes related to the aha! moment.

### 1.4. Hypotheses

In the experiment reported here, we compared aha! moments experienced during insight problem solving to those in generative ideation. We hypothesized that aha! responses (in both generative ideation and insight problem solving) have a graded structure, i.e., participants will report varying strengths of aha! moments experienced during both tasks. We also hypothesized that insight problems would be associated with stronger aha! moments because of the greater difficulty of the task and the more frequent need for restructuring compared to idea generation. We further hypothesized that aha! strength would be greater for ad hoc category generation (e.g., list all the green things you can think of), a task similar to divergent thinking, than for taxonomic category generation (e.g., list all the fruits you can think of). We also expected differences in aha! strength based on problem type, such that aha! strength would be greater for PAT problems than for CRA problems, as observed previously (Smith et al., 2025b).

As for counterbalancing, we expected that participants asked to do the category generation task first would show increased aha! strength for category generation. In contrast, had they first completed the verbal insight problems, then we would hypothesize that the aha! moments they experience serve as an anchor and thus the subsequent aha! moments in category generation to be rated as weaker in comparison. Furthermore, we hypothesized that a longer pause would be associated with stronger aha! moments during idea generation (Mori, 1996). In a previous study, we found that ideas accompanying an aha! moment were more novel and creative than those without an aha! (Smith et al., 2025a). We expected to replicate these findings and hypothesized that for idea generation prompts, more novel and creative ideas would be associated with higher aha! strength.

## 2. Materials and Methods

### 2.1. Participants

In total, 77 undergraduate students self-enrolled for this study via SONA systems in exchange for course credit. We did not collect any demographic data from the participants because we did not have any relevant hypotheses for doing so. Participants were also briefed on alternatives to participating in the study that would earn them equal credit toward the course. Eight participants were excluded from the study due to incomplete data, and five participants were excluded because they reported every response as an aha! moment for both idea generation and problem-solving tasks, regardless of whether a response was submitted. These five participants also reported every aha! moment as a “5,” showing no variability in the strength of aha! moments. This failure to follow instructions was deemed post hoc as exclusionary criteria, as was done in a previous study of problem solving (M. Becker et al., 2024). Data from 64 participants were analyzed.

### 2.2. Materials and Design

To investigate aha! moments in insight problem solving, we used two types of verbal insight problems: the Compound Remote Associates test (Bowden & Jung-Beeman, 2003) and the Polysemous Associates Test (Smith et al., 2025b). Compound Remote Associates problems (CRA) include three cue words with a single solution word, which forms a compound word or phrase with each cue. The solution word is remotely associated with the cues. An example of a CRA problem is “SLEEPING, BEAN, TRASH”, and the answer is “BAG”—sleeping bag, bean bag, and trash bag. Polysemous Associates Test (PAT) problems include two cue words, each of which is related to a distinct meaning of the solution word. An example of a PAT problem is “JEWELRY, BELL,” and the answer is “RING.” We used 32 PAT problems and 32 CRA problems of similar difficulty, all of which were used previously by Bowden and Jung-Beeman (2003) and Smith et al. (2025b). In the problem-solving task, CRA and PAT problems were presented in a fixed order, wherein they were interleaved in an ABBABAAB pattern. See Appendix A for the full set of CRA and PAT problems.

To investigate aha! moments in category generation, we used 8 category generation tasks, classified as either taxonomic categories or ad hoc categories. Taxonomic categories refer to categories for which a concrete list of members is readily available in memory; for example, names of fruits, birds, or colors. For this experiment, we used the following taxonomic category prompts: four-footed animals, fruits, metals, and countries (presented in this order). Ad hoc categories refer to categories for which a ready list of members is not available in memory, and so the members must be generated spontaneously. In this experiment, we used the following ad hoc prompts: liquids, things that make noise, things that are green, and things made of wood (presented in this order; see Appendix A).

All participants responded to all prompts across both tasks. The order of the insight problem-solving and category generation tasks was counterbalanced between participants. In condition A, the category generation tasks were presented first, and in condition B, the insight problems were presented first.

### 2.3. Measures

#### 2.3.1. Problem-Solving Measures: Accuracy and Response Time

Measures specific to problem solving included accuracy and response time. Accuracy refers to the percentage of responses that were correct. Response time refers to the time from problem presentation to the aha! click (i.e., pressing the aha! or non-aha! button).

#### 2.3.2. Category Generation Measures: Pause Time, Novelty, and Average Response Frequency

Measures specific to category generation included pause time, frequency, and novelty. Pause time refers to the time from pressing the “Enter” key on the previous response to pressing the aha! or non-aha! button. To measure average response frequency (or just ‘frequency’), the number of aha! responses and non-aha! responses in a 3 min period were averaged within subjects. We then computed a novelty score based on the between-subject occurrence of a response, calculated using the formula [(N − frequency) × 100]/(N − 1), where N represents the number of participants; thus, a response given by every participant would have a novelty score of 0, whereas a response given by only a single participant would have a novelty score of 100.

#### 2.3.3. Aha! Measure

Aha! moments were described to participants as any ideas that “pop into mind,” “come out of nowhere,” and “feel sudden, surprising, and unexpected.” When typing the solution to each problem, participants were asked to click one of two buttons: one that said “AHA!” and the other that said “Not AHA!” When participants pressed the “AHA!” button, they were subsequently asked to rate how strong the aha! moment felt on a scale from one to five, with one indicating a very weak aha! moment and five indicating a very strong aha! moment. Our use of a two-step procedure (binary decision followed by graded judgment) was intended to create a qualitative distinction between the experience of no aha! moment and the experience of a weak aha! moment.

#### 2.3.4. Aha! Strength

We measured aha! strength using a two-step method that was novel to this study. To assess aha! strength in problem solving, we asked participants for a two-step judgment of aha! after each response as follows. First, they were asked to report whether a response felt like an aha! moment (aha!) or not (not aha!). Then, if a response was reported as an aha!, we asked participants for a graded judgment of aha!, using a rating scale of 1 (very weak aha!) to 5 (very strong aha!).

To compare with the standard method of measuring aha! strength, we first used a standard measure of aha! strength, which averaged all responses, including those that were non-aha! responses (rating of 0). We also used a two-step measure of aha! strength, which only included responses reported as aha! moments.

We did not measure impasse or restructuring.

#### 2.3.5. Aha! Rate

For insight problem solving, the aha! rate refers to the average proportion of correct responses that were aha!s (excluding false aha! reports). For category generation, the aha! rate refers to the average proportion of total responses that were aha!s.

### 2.4. Procedure

Participants assembled in a quiet lab room, where they consented and were given a link that directed them to the Pavlovia webpage that hosted the experiment. Upon opening the link, participants were given instructions to read at their own pace.

First, participants were given a general description of aha! moments and instructed to report whether their solution to the problem felt like an aha! moment.

Following this, participants either saw instructions for the problem-solving portion or the category generation task, depending on the counterbalance condition. For the problem-solving task, participants were told that they would see 64 word problems in total, which would be one of two types: 32 two-word PAT problems and 32 three-word CRA problems. For both types of problems, the solution would be a single word. They would be given 15 s to read and solve each problem before the next problem was presented. If they thought of an answer, they were asked to indicate if the response felt like an aha! and rate the strength of the aha! on a scale of one (very weak aha!) to five (very strong aha!) before typing the response.

For the category generation task, participants were told that they would see a series of prompts and would receive 3 min to generate as many responses as they could think of for each prompt. They were asked to include as many unusual and imaginative things as they could think of. For each response, they were instructed to report whether their response was an aha!, rate their aha! responses on the 1–5 rating scale, and then type their response in the textbox and click “Enter.”

### 2.5. Data Preprocessing

For the category generation task, we used a mixture of automated and manual cleaning methods. Automated methods included spelling correction, string squishing and lemmatization in R (version 4.3.1). Spelling correction was done using the Microsoft Office spell-check function. String squishing involved removal of any leading or trailing spaces. Lemmatization involved making all plural responses singular and making parts of speech homogenous. For example, “sings,” “sing,” and “singing” would all become “sing”.

Manual data cleaning was conducted by one coder and reviewed by all authors of the manuscript and included some subjective assessment of validity, removal of repeated answers, and grouping of some synonyms. First, nonsense responses that did not answer the question (i.e., “I don’t know) or were incomprehensible were removed as invalid. Next, incorrect responses that did not fit the given category were removed as invalid. For example, the response “Asia” was marked as incorrect for the taxonomic list “countries.” Repeated answers from the same participant were excluded. Instances of ambiguous category membership and synonym grouping were made at the authors’ discretion. For example, “cabinet” and “cupboard” were grouped and treated as the same response for novelty scoring. The entire dataset with original and cleaned responses is available for transparency.

## 3. Results

### 3.1. Descriptive Statistics

The sample (*n* = 64) solved a moderate amount of verbal insight problems (*M* = 24.88, *SD* = 8.70), with a mean accuracy of *M* = 39.67% (*SD* = 13.86%) out of 64 problems. For solved problems, the sample reported an average of 13.41 (*SD* = 10.25) aha! moments. The average aha! rate was 52.77% (*SD* = 34.03%). See Table 1 for descriptives between PAT and CRA problems.

In category generation, the average number of responses given per 3 min cue was 15.85 (*SD* = 7.79). On average, 30.44% of responses were reported as aha! moments, although the variability was high (*SD* = 29.16%). The average pause time for a response was 5.25 s (*SD* = 1.89), and the average novelty of a response was 65.55 (*SD* = 4.73). See Table 1 for descriptives between ad hoc and taxonomic cues.

### 3.2. Task Comparison: Problem Solving vs. Category Generation

Considering the differences in task structure between problem solving and category generation, direct comparisons should be interpreted with caution. Specifically, the frequency of aha! reports in our problem-solving task had an upper bound equal to the number of problems attempted (*n* = 64) and is further restricted by the number of solved problems. In contrast, our generation task had virtually no upper limit and resulted in many more total observations per subject (*M* = 126.08, *SD* = 35.71) compared to an average of 24.88 (*SD* = 8.70) solved CRA and PAT problems.

#### 3.2.1. Aha! Rate Comparison

**Figure 1:**
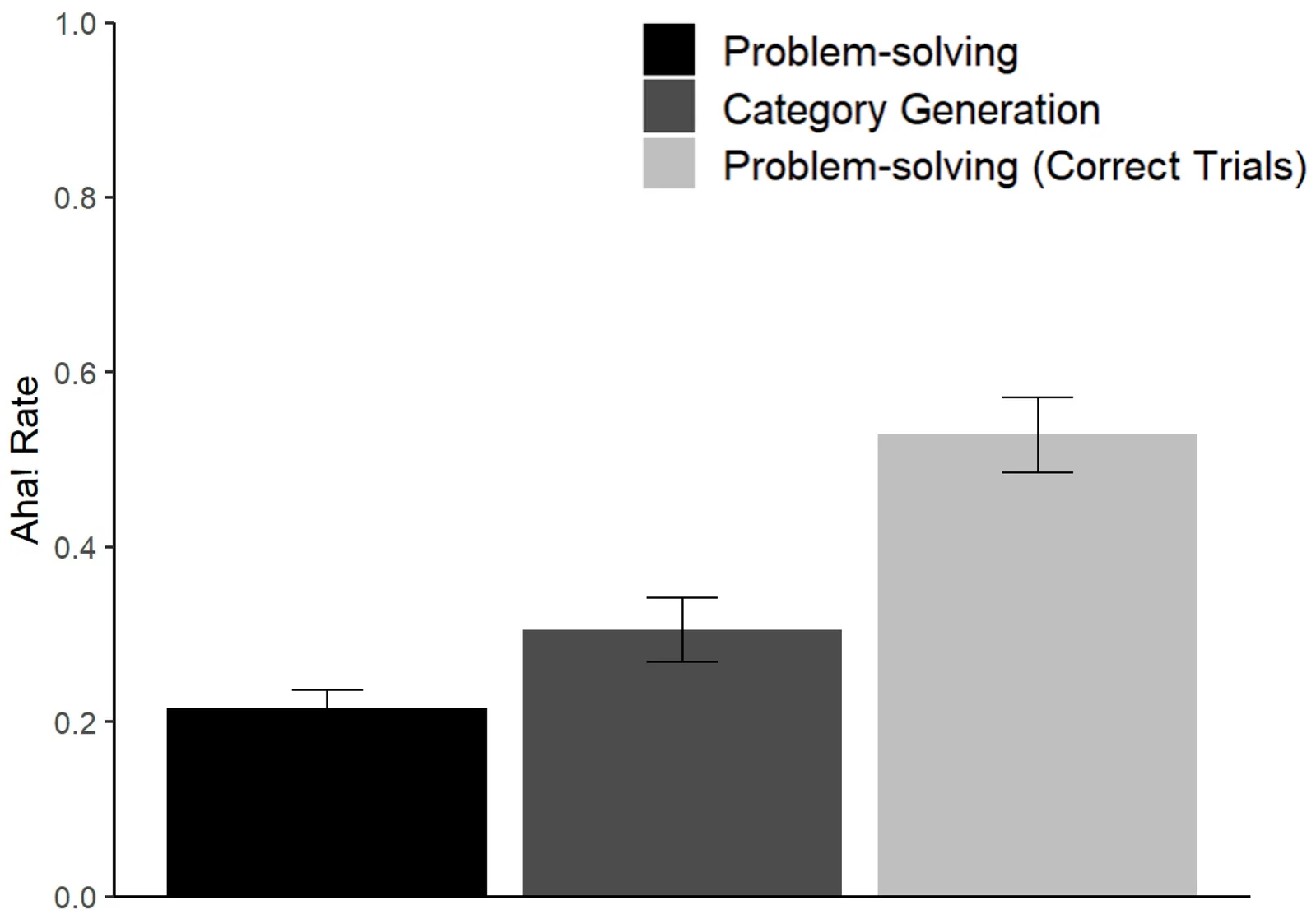
Aha! Rate by Trial Type.

A within-subject *t*-test revealed a significant difference, *t*(63) = 2.72, *p* = .008, between the aha! rate reported for problem solving (*M* = 21.54%, *SD* = 16.53%) and category generation (*M* = 30.44%, *SD* = 29.16%). However, the aha! rate for correct problem-solving trials, i.e., excluding false aha! reports, (*M* = 52.77%, *SD* = 34.04%) was significantly higher than the aha! rate observed in category generation, *t*(63) = 6.41, *p* < .001; see Figure 1.

#### 3.2.2. Standard Aha! Strength Comparison

The standard measure of aha! strength includes both aha! (1–5) and non-aha! responses (scored as 0 strength) aggregated for each subject across each task. There was a significant positive correlation, *r*(62) = 0.58, *p* < .001, between mean strength ratings in problem solving and category generation.

A mixed 2 (task) × 2 (counterbalance) ANOVA was computed to compare within-subject differences of task and between-subject effects of task order. There was no main effect observed for task type, *F*(1, 62) = 1.93, *p* = .170, *η_p_*^2^ = 0.03, between category generation (*M* = 1.03, *SE* = 0.15) and problem solving (*M* = 0.87, *SE* = 0.09). There was no effect of counterbalance, *F*(1, 62) = 0.06, *p* = .805, *η_p_*^2^ < 0.001. There was no difference in standard aha! strength when problem solving was completed first (*M* = 0.98, *SE* = 0.12) compared to category generation first (*M* = 0.92, *SE* = 0.13). Finally, there was no significant interaction observed, *F*(1, 62) = 0.77, *p* = .383, *η_p_*^2^ = 0.01; see Table 2.

To provide evidence supporting the null effects, Bayes factors (*BF*_10_) were estimated using the “BayesFactor” package in R (Morey & Rouder, 2026) and assumed default priors. The effect of task type provided anecdotal support for the null hypothesis, which was 2.17 times as likely (*BF*_01_) than under the alternative hypothesis. The effect of counterbalancing was 3.23 times more likely (*BF*_01_) under the null hypothesis, and the full model, including the interaction, was 7.14 times more likely (*BF*_01_) under the null. See Table 2 for *BF*_10_ estimates.

#### 3.2.3. Two-Step Aha! Strength Comparison

The same analyses were conducted to compare the mean two-step aha! strength, that is, excluding all non-aha! responses. Some subjects (*n* = 4) did not report any aha! responses for either task and were thus excluded from this analysis. There was a significant positive correlation, *r*(58) = 0.53, *p* < .001, between mean strength ratings in problem solving and category generation.

The mixed 2 (task type) × 2 (counterbalance) ANOVA observed no significant main effects for task, *F*(1, 58) = 0.01, *p* = .943, *η_p_*^2^ < 0.001, or task order, *F*(1, 58) = 0.01, *p* = .937, *η_p_*^2^ < 0.001. Results suggest no detectable difference in reported aha! strength between problem solving (*M* = 3.04, *SE* = 0.11) and category generation (*M* = 3.04, *SE* = 0.11) when rounded to two decimal places. There was no difference in strength when problem solving was completed first (*M* = 3.03, *SE* = 0.10) compared to category generation first (*M* = 3.05, *SE* = 0.11). There was no significant interaction effect observed, *F*(1, 58) = 0.64, *p* = .426, *η_p_*^2^ = 0.01.

To provide evidence supporting the null effects, Bayes factors (*BF*_10_) were estimated using the “BayesFactor” package in R and assumed default priors. The effect of task type provided moderate support for the null hypothesis, which was 5.26 times more likely (*BF*_01_) than under the alternative hypothesis. The effect of counterbalancing was 3.23 times more likely (*BF*_01_) under the null hypothesis, and the full model, including the interaction, was 16.66 times more likely (*BF*_01_) under the null. See Table 2 for *BF*_10_ estimates.

### 3.3. Problem-Solving Analyses

There was no significant within-subjects difference in accuracy, *t*(63) = 0.10, *p* = 0.918, between CRA (*M* = 39.6%, *SD* = 13.1%) and PAT (*M* = 39.8%, *SD* = 19.5%) problems. There was a significant difference in aha! rates (i.e., the percentage of correct responses reported aha!), *t*(63) = 2.86, *p* = .006, with CRA (*M* = 55.0%, *SD* = 35.5%) outperforming PAT (*M* = 48.4%, *SD* = 36.8%) problems.

**Figure 2:**
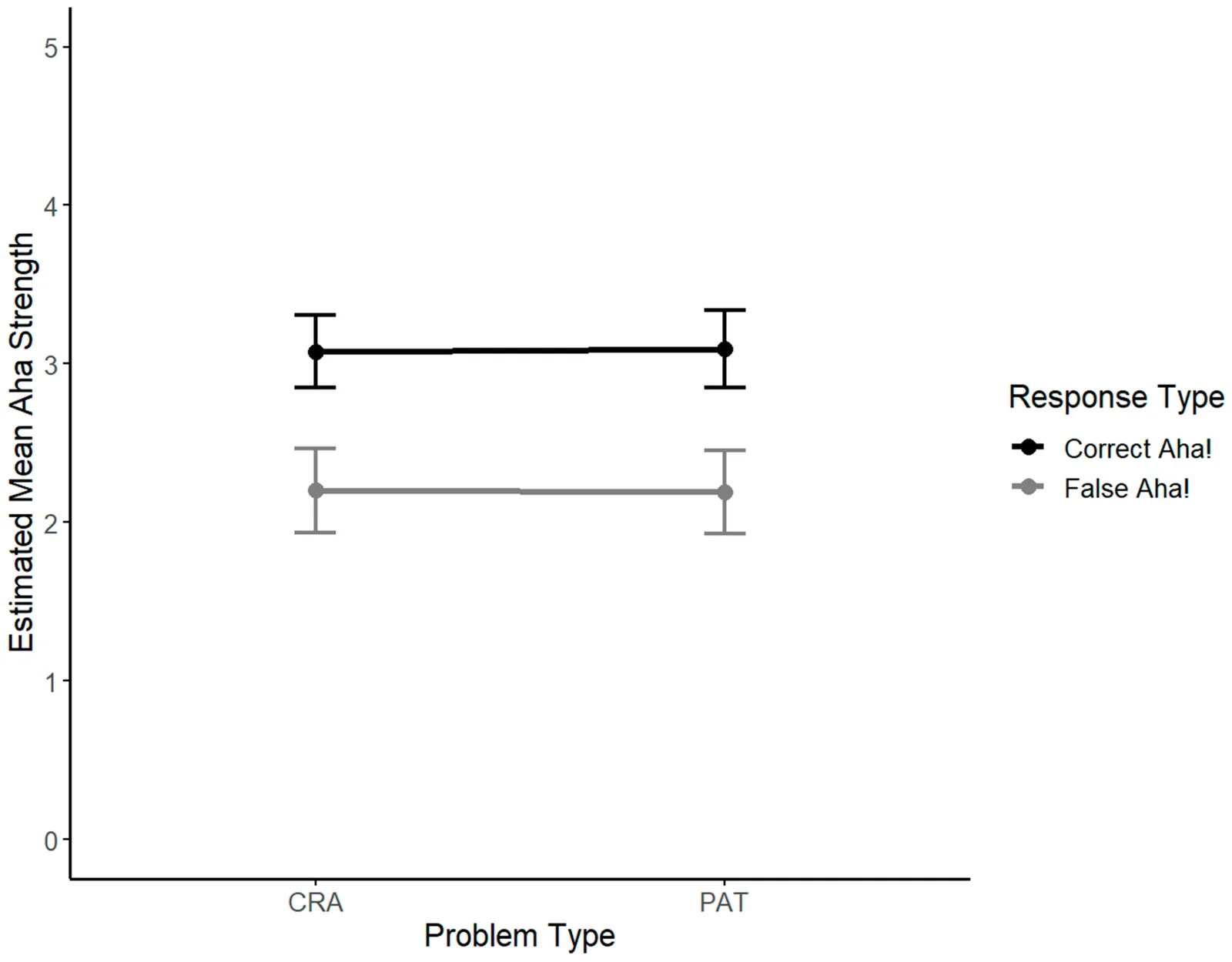
Aha! strength ratings for correct and incorrect responses in problem-solving.

There was a strong, positive correlation between aha! strength scores for CRA and PAT problems, *r*(62) = 0.85, *p* < .001. Furthermore, a 2 × 2 mixed effects model was calculated to examine aha! strength differences between problem type (CRA vs. PAT) and response type (false aha! vs. correct aha!) with random effects for participants. There was no significant difference between CRA and PAT problems (*p* = .909), and no significant interaction (*p* = .906). There was a significant effect of response type (*p* < .001), such that correct aha! responses (*M* = 3.09, *SE* = 0.12) had higher strength ratings than false aha! responses (*M* = 2.20, *SE* = 0.13); see Figure 2.

### 3.4. Category Generation Analyses

There was no significant difference in the two-step aha! strength, *t*(59) = −1.21, *p* = .230, between ad hoc (*M* = 3.22, *SD* = 0.94) and taxonomic (*M* = 3.13, *SD* = 0.99) cues. There was no difference between taxonomic and ad hoc cues for total response frequency, *t*(63) = 1.44, *p* = .155, or aha! rate, *t*(63) = 0.84, *p* = .406, between cue types.

**Figure 3:**
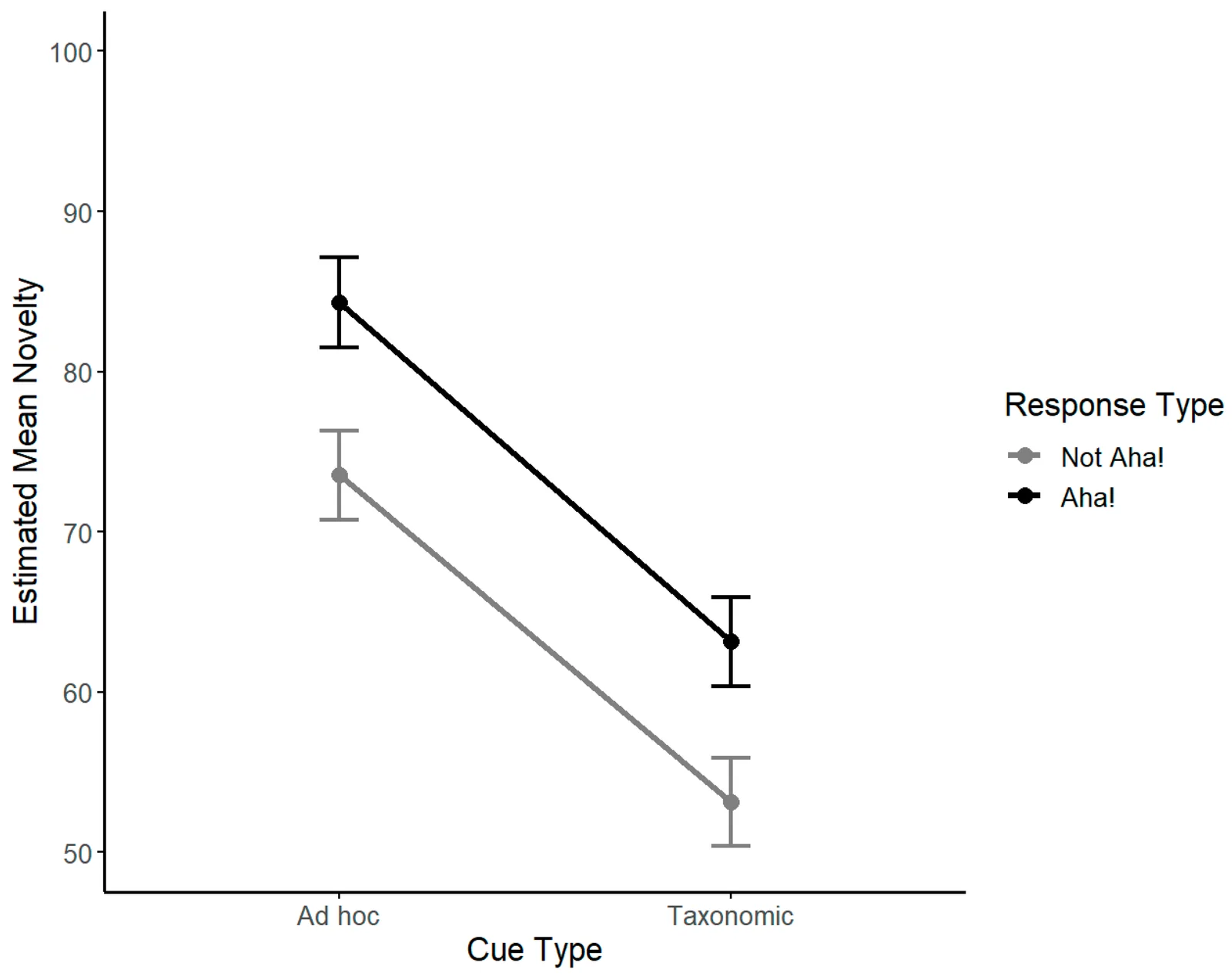
Novelty scores by response type and cue type.

A 2 × 2 mixed effects model was calculated to examine mean novelty differences between cue type (taxonomic vs. ad hoc) and response type (aha! vs. not aha!) with random effects for participants. The main effects of response type and cue type were both significant (*p* < .001); aha! responses (*M* = 73.70, *SE* = 1.04) were significantly more novel than non-aha! responses (*M* = 63.30, *SE* = 1.04). Ad hoc responses were significantly more novel than taxonomic. The interaction was not significant. See Figure 3 below.

#### Time Course Analyses

To analyze within-subject differences in the time course of responding for a 3 min period of generation, we aggregated participant data across six 30 s intervals (i.e., first 30 s to sixth 30 s). Standard aha! strength was positively correlated with novelty, *r*(319) = 0.46, *p* < .001); however, the effect was weaker with two-step aha! strength, *r*(248) = 0.15, *p* = .014. Standard aha! strength also had a positive association with pause time, *r*(319) = 0.46, *p* < .001, and 30 s interval, *r*(319) = 0.51, *p* < .001. The effect for two-step aha! strength was again weaker for both pause time, *r*(248) = 0.27, *p* < .001, and 30 s interval, *r*(248) = 0.21, *p* = .001.

**Figure 4:**
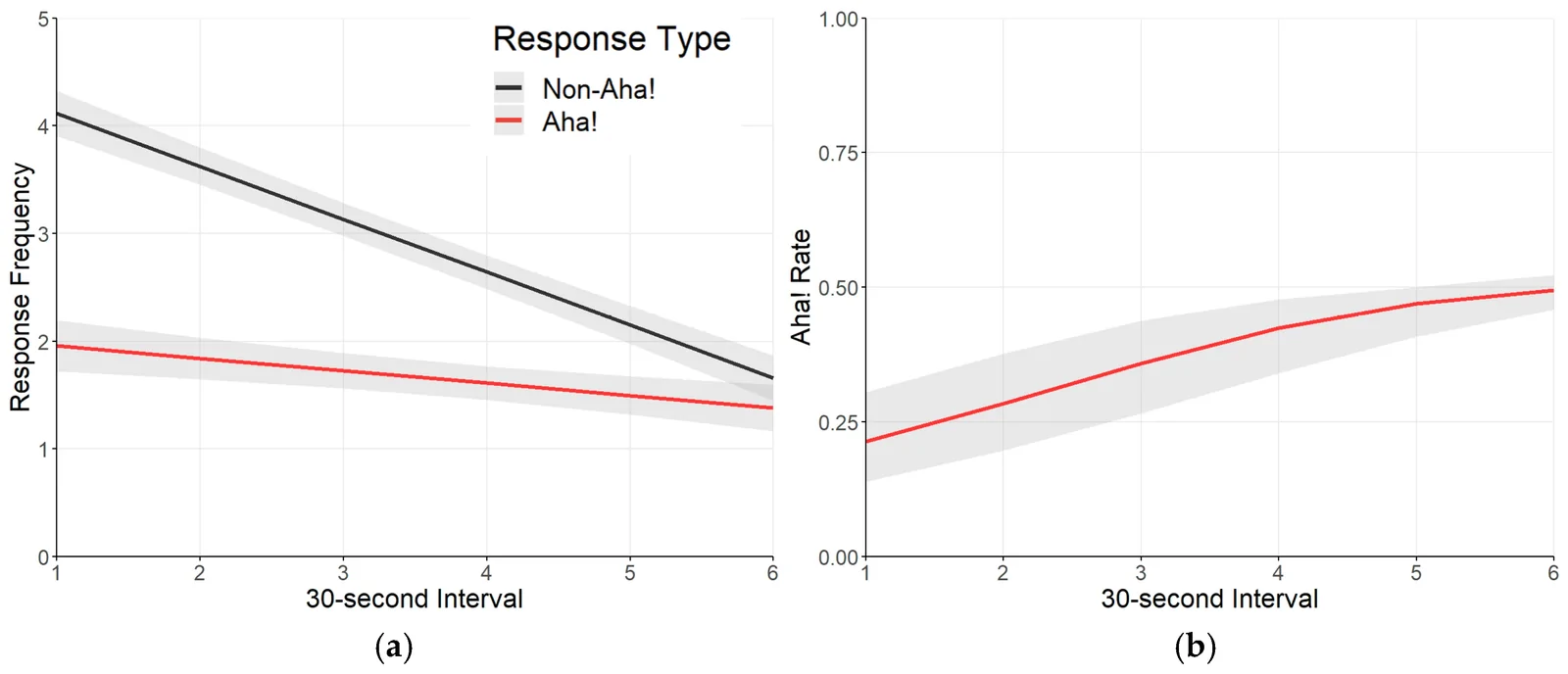
(**a**) Predicted frequency of aha! and non-aha! responses over 3 min generation period. (**b**) Predicted aha! rate over 3 min generation period.

A mixed effects linear regression was conducted to predict response frequency with fixed effects interval, response type (aha! or not aha!), along with their interaction and included random effects for participants and cue type (taxonomic or ad hoc). There was a significant difference (*b* = −2.54, 95% CI [−2.72, −2.36]) in response type; aha! frequency was lower than non-aha! frequency overall. Interval was a significant predictor (*b* = −0.87, 95% CI [−0.94, −0.80]) indicating that response fluency decreased linearly over the 3 min period. The interaction between response type and interval was significant (*b* = 0.38, 95% CI [0.34, 0.42]). Consistent with prior findings, response frequency decreased throughout generation, but the frequency of non-aha! responses decreased at a significantly greater rate; see Figure 4a. The full model explained a large amount of variance in response fluency (marginal *R*^2^ = 0.41, conditional *R*^2^ = 0.53).

To predict the probability of an aha! response occurring, a mixed effects logistic regression was conducted and included fixed effects for 30 s interval, pause time, novelty, and response fluency, with random effects for participants and cue type (taxonomic or ad hoc). The model demonstrated good fit, BIC = 5554.4, compared to an intercept-only model, BIC = 6623, although random effects accounted for a large portion of variance (marginal *R*^2^ = 0.16, conditional *R*^2^ = 0.64). Aha! responses were significantly more likely to occur in later intervals of generation (β = 0.70, 95% CI [0.62, 0.78]), after longer pauses (β = 0.46, 95% CI [0.39, 0.54]), with higher novelty (β = 0.21, 95% CI [0.12, 0.30]), and lower response fluency (β = −0.61, 95% CI [−0.72, −0.50]). See Figure 4b.

## 4. Discussion

The objective of our study was to compare aha! moments reported during idea generation with those reported during problem solving. To this end, we used a two-step approach to measure aha! moments, in which we used a binary (yes/no) judgment of aha!, followed by a graded measure of the strength (intensity) of the aha! on a scale of 1 (very weak aha!) to 5 (very strong aha!). Our results replicated the findings from Smith et al. (2025a), in which participants reported aha! moments during the category generation tasks. We interpret our findings to infer an aha-like experience rather than a classical insight report that entails impasse and restructuring.

We were interested in the potential differences in aha! strength between category generation and problem-solving tasks. Given that aha! moments are well-established during CRA and PAT problems (Bowden & Jung-Beeman, 2003; Smith et al., 2025b), we expected an anchoring effect when participants completed the problem-solving task first. We hypothesized that if aha! moments reported during category generation are unlike those reported during problem solving, then experiencing aha! moments during problem solving would calibrate participants to what an aha! is supposed to feel like, resulting in lower aha! strength in the subsequent category generation task. We used a unique, two-step approach for soliciting aha! judgments, in which participants first reported a binary aha! or not aha!, followed by a graded judgment of strength if an aha! was reported. Using the two-step method, we observed no within-subject difference in aha! strength between problem solving and category generation, even when the task order was counterbalanced. Irrespective of whether the problem-solving task was done before or after category generation, participants did not report the strength of the aha! moment differently. Furthermore, Bayesian analyses provided strong evidence for the null hypothesis, using both the standard measure and the two-step measure to compare aha! strength between the task types.

This finding is surprising because aha! strength during problem solving has been associated with the feeling of certainty about a solution, where certainty is defined as confidence in the correctness of a solution (Danek et al., 2014; Danek & Wiley, 2017). In contrast, the category generation task does not demand a single correct solution—it instead entails generating a series of solutions that meet a criterion, emerging from iterative generative and exploratory processes (Finke et al., 1992). We did not measure certainty in our sample, but the nature of the category generation task asks for multiple responses that meet the constraints of the category presented to the participants rather than one solution that feels certain or correct. Our participants were instructed to list as many unusual and imaginative ideas as they could think of, which means there may have been some relaxation in the constraints defining a category (Ward, 1994). For example, participants listed mythical creatures such as unicorns when listing four-footed animals. Our study shows that later ideas were more novel, and that the novelty of ideas was positively correlated with aha! strength. These findings point to two important ideas—first, that aha! moments are observed during tasks that are not categorically insight problems, and, second, that aha! moments of varying intensities occur even when participants are searching for ideas with different goals in mind (i.e., searching for a correct solution to a problem as opposed to searching for multiple, possibly creative members of a category that lie on a continuum of correctness).

We also found that there was no difference in aha! strength between CRA and PAT problems, further supporting the idea that problem type may not be the most important determinant of aha! intensity. Previous studies support this; for example, Webb et al. (2018) found that there was no significant difference in average aha! ratings between traditional insight problems and non-insight problems when accounting for correct solutions.

Whereas we found a higher aha! rate for correct problem-solving solutions compared to category generation ideas, these findings may be accounted for by the difference in the total number of observations. Participants listed as many ideas as they could think of during category generation, but only one solution during problem solving, resulting in a higher total number of responses for category generation. When correctly solved, aha! moments are more likely to be reported during problem solving, but when accounting for all responses (correct and incorrect), there is no difference in the rate at which participants report aha! moments.

These findings tie into the growing body of literature that shows aha! moments occur not just during convergent thinking tasks, but also during divergent thinking tasks (Smith et al., 2025a; Ding et al., 2023; Yu et al., 2024). This is interesting because it warrants future structured investigation of whether insight problem solving and idea generation share similar underlying retrieval dynamics, as proposed by the unified theory of idea generation (Smith et al., 2025a).

For idea generation, we replicated previous findings (Smith et al., 2025a) that showed a serial order effect, where frequency of responses decreased over time and later ideas were more novel, had longer inter-response times (pause time), and were more likely to be reported as aha! moments. A novel finding from our study was that stronger aha! moments were reported for later ideas compared to early ideas.

What makes later ideas accompany stronger aha!s during idea generation? During problem solving, suddenness is typically associated with stronger aha! reports (Danek & Wiley, 2017). The sudden flooding of an idea into consciousness, wherein a response follows a switch from a low fluency state to a high fluency state, has been thought to underlie some aha! moments (Topolinski & Reber, 2010). This sudden and unexpected idea after a long period of unsuccessful search may be reported as a stronger aha! when preceded by a longer search, as shown by the longer pauses before an aha! during category generation.

However, there is reason to suspect that suddenness alone is not a comprehensive explanation of this finding. A study showed an incubation effect during category generation, where alternating between generating members of ad hoc and sense impression categories was associated with higher novelty and frequency of ideas (Smith et al., 2017). They attributed these findings to the idea that output interference occurs during category generation, wherein retrieving an idea from the search space biases it to become more likely to be retrieved again when using the same retrieval cues (e.g., Raaijmakers & Shiffrin, 1981; Rundus, 1973). This diminishes the probability of retrieving other yet-unretrieved ideas, but alternating between categories mitigates the effect of output interference by forgetting fixation (Smith et al., 2017). They also speculated that restructuring occurs during category generation because the incubation effect was limited to ad hoc and sense categories, which are more flexible than taxonomic categories. Other studies show that participants retrieve ideas in similar clusters (Ovando-Tellez et al., 2022). When the most accessible ideas in a cluster are exhausted, participants tend to pause for longer and then switch to another cluster, consistent with the foraging theory in semantic search (Hills et al., 2015). S. Acar and Runco (2017) found that response latency (pause time, or inter-response time) is 5 s higher before category switching during divergent thinking, and a follow-up study showed that response latency was also predictive of originality (O. A. Acar et al., 2019). Our results were consistent with these accounts; thus, it is possible that aha! moments in our study may have been preceded by restructuring. However, more rigorous scrutiny of this claim would entail manipulations of either restructuring, incubation, or forgetting fixation, which our study did not measure.

We defined aha! moments to our participants as ideas that seemed to “pop into mind”, “come out of nowhere”, and feel “sudden and unexpected”. Given this definition, and the fact that we did not measure fixation, we speculate that these aha! reports may resemble mind pops, which are spontaneous recollections of semantic, episodic or autobiographical memory (e.g., Kvavilashvili & Mandler, 2004; Mandler, 1995). Flashes of insight are understood as a type of spontaneous thought (Fox & Christoff, 2018), much like daydreaming and mind-wandering (Fox & Christoff, 2018), although there is limited study on whether mind-pops characterize insight or aha! moments.

Another alternative explanation for our findings may be participants’ interpretation of the instructions, or an expectancy effect wherein participants may have reported aha! moments because they were expecting to have aha! moments. However, there is evidence to suggest that our findings are not fully explained by this argument (Smith et al., 2025a). If the aha! moments were reported due to participants’ interpretation of the instructions, then we would expect aha! reports on both tasks to be confounded by the instructions. However, this was not observed—our findings for the problem-solving task and the idea generation task are consistent with previous studies. Aha! moments during problem solving are associated with several characteristics that distinguish aha! solutions from non-aha! solutions, such as better memory for aha! solutions (Danek et al., 2013; Kizilirmak et al., 2016), higher accuracy of aha! solutions (Danek & Salvi, 2020; Salvi et al., 2016; Salvi & Bowden, 2024), and a longer pause before an aha! moment (e.g., Schooler & Melcher, 1995; Smith et al., 2025a). In our study, we used CRA and PAT problems, both of which have reliably produced aha! moments (Bowden & Jung-Beeman, 2003; Smith et al., 2025b). We replicated the aha! accuracy effect (Danek et al., 2014, Danek & Wiley 2017) for the problem-solving task, where correct aha! solutions were rated as stronger than false-aha solutions. During idea generation, aha! ideas appeared to be qualitatively different from non-aha! ideas because they were accompanied by higher novelty than non-aha! ideas. Furthermore, aha! strength was positively correlated with novelty. Together, these distinctive characteristics of aha! solutions and ideas suggest a qualitative difference between aha! and non-aha! moments and are unlikely to be accounted for by expectancy effects alone.

An important contribution to our study is the use of a two-step reporting method for aha! moments. We used a binary judgment first, followed by a graded judgment of the intensity of the aha! on a scale of 1 (very weak aha!)–5 (very strong aha!). Previous studies use a continuous scale that starts at 0 (no aha!) instead of 1 (weak aha). This is an important distinction because of the implication that the absence of an aha! lies on the same continuum as the presence of a weak aha!. This issue has been discussed previously in related work of graded metacognitive judgments, such as in the feeling-of-knowing (Meunier-Duperray et al., 2025; Rahnev, 2025) and recognition memory (Rotello et al., 2005; Wixted & Mickes, 2010; Yonelinas, 1994) literature. It is important to consider that continuous rating scales can conflate the absence of evidence with low-strength evidence, whereas two-step procedures (binary decision followed by graded judgment) preserve a qualitative distinction between no experience and a weak experience. We found that aha! strength comparisons of novelty and pause time using the two-step measure resulted in smaller effect sizes compared to the standard measure. We speculate this could be because the baseline difference in novelty and pause time between aha! and non-aha! responses may be larger than the difference in novelty and pause time across levels of aha! strength. This inclusion of non-aha! responses (i.e., use of the standard 0–5 scale) may be inflating the effect sizes observed during aha! strength analyses. Our use of a two-step report is a novel methodological contribution to graded judgments of insight.

Our two-step aha! report method may also allow a better understanding of how the subjective experience of insight is associated with psychophysiological measures of insight. Studies using pupillometry have reported a noticeable increase in pupil size when participants reported aha! moments (Van der Wel & Van Steenbergen, 2018; J. Becker et al., 2024; Einhäuser et al., 2008). Salvi et al. (2020) found that pupil diameter increased in 60.5% of trials solved by insight, peaking about 200 ms before the aha! moment. Our two-step approach, coupled with the category generation task, may be used to determine if aha! strength is associated with the diameter of the pupil during dilation, which could facilitate investigations into the role of effort (Van der Wel & Van Steenbergen, 2018), emotional arousal (Bradley et al., 2008), and reward (Schneider et al., 2018; Fröber et al., 2020) in the phenomenology of insight.

We found a few within-task differences. During category generation, we found that responses to ad hoc category cues were more novel than responses for taxonomic category cues. This is consistent with the previous literature (Smith et al., 2017, 2025a). Smith et al. (2025a) also reported that divergent thinking responses (Alternative Uses Test responses) were significantly more novel than both taxonomic and ad hoc category responses. Therefore, it is important to assess how aha! moments reported during divergent thinking responses might (or might not) differ from those reported during problem solving. We also found that CRA problems had a higher proportion of aha! reports compared to PAT problems. This is different from findings reported by Smith et al. (2025b). These differences may be explained by the choice of PAT problems—the solution rates for the PAT problems chosen for this study were lower than those from the previous study, which may explain the lower aha! rate.

We also observed high variability in aha! rates and aha! strength, which may reflect underlying individual differences in how participants report and experience aha! moments. However, we found strong positive correlations in aha! strength between problem-solving and category generation (using the standard and two-step measures), suggesting that these individual differences were stable across task types. Nevertheless, further investigation is necessary to determine the factors that affect individual differences in aha! strength.

Our study has a few limitations. Whereas we counterbalanced the order of task presentation, we presented the items in each task in a fixed, interleaved pattern, which may introduce some fatigue effects. In addition, scoring responses for novelty entailed some manual decisions about synonym grouping and determining invalid responses. Our study used one rater for these decisions, which may introduce a subjective variable for replication. We have detailed the scoring process and included our scored data to facilitate replication of this study.

Our findings help to contextualize aha! moments as a characteristic of the idea generation process by showing that the aha! experience is largely similar in terms of strength of the aha!, irrespective of whether the goal of the ideation process is to arrive at a single correct solution, or to brainstorm multiple ideas.

## Figures and Tables

**Table 1:** Descriptive statistics for problem solving and category generation.

Problem-Solving Measure ^1^	CRA (*SD*)	PAT (*SD*)
Problems Solved	*M* = 12.41 (4.04)	*M* = 12.47 (6.10)
False Aha! Reports	*M* = 2.13 (2.72)	*M* = 2.73 (3.67)
Correct Aha! Reports	*M* = 6.95 (5.13)	*M* = 6.45 (5.81)
Response Time ^2^	*M* = 6.27 (2.84)	*M* = 6.45 (5.81)
Standard Aha! Strength	*M* = 0.89 (0.75)	*M* = 0.85 (0.75)
Two-step Aha! Strength	*M* = 3.09 (0.94)	*M* = 3.10 (0.82)
**Category Generation Measure ^3^**	**Taxonomic (*SD*)**	**Ad Hoc (*SD*)**
Non-Aha! Responses	*M* = 11.51 (9.70)	*M* = 11.36 (8.29)
Aha! Responses	*M* = 4.09 (4.57)	*M* = 4.75 (5.09)
Novelty	*M* = 52.14 (11.54)	*M* = 75.15 (8.36)
Pause Time	*M* = 5.06 (2.01)	*M* = 5.63 (2.47)
Standard Aha! Strength	*M* = 1.05 (1.20)	*M* = 1.03 (1.20)
Two-step Aha! Strength	*M* = 3.07 (0.84)	*M* = 2.96 (0.91)

^1^ Sample (*n* = 64) averages across 32 trials of each problem type. ^2^ Response time (seconds) for correct problem-solving trials only. ^3^ Sample (*n* = 64) averages for a 3 min generation trial.

**Table 2:** Mean Strength Comparisons and Bayes Factors (BF_10_).

Standard Aha! Strength ^1^	Mean (*SE*)	Bayes Factor
Task Type		*BF*_10_ = 0.46
Problem Solving (A) ^2^	*M* = 0.95 (0.13)	
Category Generation (A)	*M* = 1.01 (0.21)	
Counterbalance		*BF*_10_ = 0.31
Problem Solving (B)	*M* = 0.79 (0.13)	
Category Generation (B)	*M* = 1.06 (0.21)	
Interaction		*BF*_10_ = 0.14
**Two-step Aha! Strength ^3^**		
Task Type		*BF*_10_ = 0.19
Problem Solving (A)	*M* = 3.08 (0.15)	
Category Generation (A)	*M* = 2.99 (0.15)	
Counterbalance		*BF*_10_ = 0.31
Problem Solving (B)	*M* = 3.01 (0.15)	
Category Generation (B)	*M* = 3.09 (0.15)	
Interaction		*BF*_10_ = 0.06

^1^ Standard aha! strength is measured as the average strength rating (0–5) of all responses, where non-aha! and incorrect responses have a strength of 0 from (*n* = 64) subjects. ^2^ The counterbalance conditions are denoted (A) problem solving was completed first and (B) category generation was completed first. ^3^ Two-step aha! strength is measured as the average strength rating (1–5) of only aha! responses from (*n* = 60) subjects.

## Data Availability

The original data presented in this study is openly available in FigShare (https://doi.org/10.6084/m9.figshare.32048760).

## Abbreviations

The following abbreviations are used in this manuscript:

PAT	Polysemous Associates’ Test
CRA	Compound Remote Associates
RAT	Remote Associates’ Tests
DMN	Default Mode Network
ECN	Executive Control Network

## Appendix A

**Table A1:** The 64 Compound Remote Associates (CRA) and Polysemous Associates Test (PAT) problems, in the order in which they were presented to participants.

#	Problem	Solution	Type
1	AUTUMN PLUMMET	FALL	PAT
2	QUACK CROUCH	DUCK	PAT
3	LOSER THROAT SPOT	SORE	CRA
4	SHOW LIFE ROW	BOAT	CRA
5	DEW COMB BEE	HONEY	CRA
6	ROCKING WHEEL HIGH	CHAIR	CRA
7	GIFT NOW	PRESENT	PAT
8	FOUNTAIN BAKING POP	SODA	CRA
9	PRESERVE RANGER TROPICAL	FOREST	CRA
10	CRY SHRED	TEAR	PAT
11	AID RUBBER WAGON	BAND	CRA
12	RIBBON ARROW	BOW	PAT
13	FLAKE MOBILE CONE	SNOW	CRA
14	CRACKER FLY FIGHTER	FIRE	CRA
15	RECLINE FIB	LIE	PAT
16	CANE DADDY PLUM	SUGAR	CRA
17	SAFETY CUSHION POINT	PIN	CRA
18	DREAM BREAK LIGHT	DAY	CRA
19	FISH MINE RUSH	GOLD	CRA
20	POLITICAL SURPRISE LINE	PARTY	CRA
21	MEASURE WORM VIDEO	TAPE	CRA
22	LEFT CORRECT	RIGHT	PAT
23	DICTATOR MEASURE	RULER	PAT
24	BURST SODA	POP	PAT
25	BUG JET	FLY	PAT
26	GUITAR STONE	ROCK	PAT
27	SENSE COURTESY PLACE	COMMON	CRA
28	PIECE MIND DATING	GAME	CRA
29	WORM SHELF END	BOOK	CRA
30	QUICK STARVE	FAST	PAT
31	FLOWER FRIEND SCOUT	GIRL	CRA
32	VOWEL MAIL	LETTER	PAT
33	PIE LUCK BELLY	POT	CRA
34	PRINT BERRY BIRD	BLUE	CRA
35	CADET CAPSULE SHIP	SPACE	CRA
36	OPERA HAND DISH	SOAP	CRA
37	DATE ALLEY FOLD	BLIND	CRA
38	DIAMOND BELL	RING	PAT
39	STICK MAKER POINT	MATCH	CRA
40	FUR RACK TAIL	COAT	CRA
41	TIN ABLE	CAN	PAT
42	CARESS VETERINARIAN	PET	PAT
43	DEPARTS TWIGS	LEAVES	PAT
44	OAR COLUMN	ROW	PAT
45	CUB TOLERATE	BEAR	PAT
46	FOX MAN PEEP	HOLE	CRA
47	HOUND PRESSURE SHOT	BLOOD	CRA
48	SLEEPING BEAN TRASH	BAG	CRA
49	STUMBLE TRAVEL	TRIP	PAT
50	KNEAD MONEY	DOUGH	PAT
51	TIMEPIECE OBSERVE	WATCH	PAT
52	HOME SEA BED	SICK	CRA
53	KEYBOARD VARIETY	TYPE	PAT
54	WHEEL TALKED	SPOKE	PAT
55	BREEZE TWIST	WIND	PAT
56	MAIN SWEEPER LIGHT	STREET	CRA
57	BOOT SUMMER GROUND	CAMP	CRA
58	TELLER RIVER	BANK	PAT
59	FIG CALENDAR	DATE	PAT
60	CHILLY GROOVY	COOL	PAT
61	TINY HOUR	MINUTE	PAT
62	DUNE ABANDON	DESERT	PAT
63	LIGHT TULIP	BULB	PAT
64	RIB TREASURE	CHEST	PAT

**Table A2:** The 8 taxonomic and ad hoc category generation prompts, in the order in which they were presented to participants.

#	Prompt	Type
1	Four-footed animals	Taxonomic
2	Fruits	Taxonomic
3	Metals	Taxonomic
4	Countries	Taxonomic
5	Liquids	Ad hoc
6	Things that make noise	Ad hoc
7	Things that are green	Ad hoc
8	Things made of wood	Ad hoc
